# Matrix effect on surface-catalyzed photolysis of nitric acid

**DOI:** 10.1038/s41598-018-37973-x

**Published:** 2019-03-13

**Authors:** Chunxiang Ye, Ning Zhang, Honglian Gao, Xianliang Zhou

**Affiliations:** 1 Beijing Innovation Center for Engineering Science and Advanced Technology, State Key Joint Laboratory for Environmental Simulation and Pollution Control, Center for Environment and Health, and College of Environmental Sciences and Engineering, Peking University, Beijing, 100871 China; 2 Wadsworth Center, New York State Department of Health, Albany, NY 12201 USA; 3 Department of Environmental Health Sciences, State University of New York, Albany, NY 12201 USA

## Abstract

Photolysis rate constant of HNO_3_ on the surface (HNO_3(s)_) has been found to be enhanced by 1–4 orders of magnitude from that of gaseous HNO_3_, with HONO and NO_2_ as the main products. Such Re-NOx-ification pathway extends the apparent lifetime of reactive nitrogen species and modifies the atmospheric oxidative capacity along its long-rang transport. Despite of its importance, the detailed kinetics and mechanisms of HNO_3(s)_ photolysis are still not clear. Surface film of HNO_3_ and organic compounds is ubiquitous in the environment and imposes matrix effect on HNO_3(s)_ photolysis. Here we studied photolysis of HNO_3_ on Pyrex glass in a photochemical flow reactor over a wide range of HNO_3_ surface density (*D*_*HNO3*_) with or without the presence of model organic compounds. The photolysis rate constant of HNO_3(s)_ varied with *D*_*HNO3*_ and surface-catalysis mechanism was proposed. Organic compounds further enhance the photolysis rate constant by up to one order of magnitude via both photosensitization and H-donating reaction. The H-donating reaction enhances as well the secondary HONO yield from reaction between the primary product NO_2_ and adjacent H-donor, and thus increases the HONO/NO_2_ production ratio. Finally, detailed mechanisms involving surface-catalyisis, photosensitization and H-donating reactions was integrated.

## Introduction

Formation of gaseous nitric acid and particulate nitrate (hereafter both referred to as HNO_3_), followed by its deposition on ambient surfaces, has been traditionally considered to be the permanent removal of nitrogen oxides (NO_x_ = NO + NO_2_) from the troposphere^1^. However, increasing number of studies have demonstrated that photolysis rate constant of HNO_3_ on ambient surfaces and in aerosol particles, referred to as HNO_3(s)_, is enhanced by 1–4 orders of magnitude^2–11^, compared to that of nitric acid in the gas phase, with HONO and NO_2_ as the main products^2–5,8,9,11^. As such, cycling of HNO_3(s)_ to reproduce HONO and NO_2_ is competitive to its deposition removal, which results in a longer apparent lifetime and farer transport distance of NO_x_ in the atmosphere than originally expected. HONO and NO_2_ are known as precursors of hydroxyl radical (OH) and ozone (O_3_), respectively. Production of HONO and NO_2_ by photolysis of HNO_3(s)_ have a great impact on the atmospheric oxidative capacity, both for the polluted high-NO_x_ environments^5,10^ and the remote low-NO_x_ environments^2–4,12^.

The reported photolysis rate constant of HNO_3(s)_ varies over a range of three orders of magnitude^2–13^. For example, several laboratory studies have reported photolysis rate constants of HNO_3(s)_ of 2.2 × 10^−5^ s^−1^, 2.0 × 10^−5^ s^−1^ and 7.7 × 10^−4^ s^−1^ on silicon, glass, and sapphire surfaces^6–9^. The photolysis rate constant of HNO_3(s)_ in urban grime was found to be as high as 1.2 × 10^−3^ s^−1^ under dry condition^5^. We have studied various natural and artificial surfaces in a previous paper and measured the photolysis rate constant of HNO_3(s)_ in the range from 9 × 10^−6^ s^−1^ to 3.7 × 10^−4^ s^−1^, depending on the types of surfaces^3^.

The surface-enhanced and highly-varied photolysis rate constant of HNO_3(s)_ could be, at least in part, rationalized by the underlying micro-mechanisms^14–18^. The interaction of HNO_3(s)_ with surface reactive sites or associated molecules in the surface matrix, such as H_2_O, organic compounds, and HNO_3(s)_ itself, has the potential to distort its molecular structure^14–18^. The distorted molecular structure of HNO_3(s)_ is believed to be responsible for the “red shift” and the cross section enhancement of its absorption spectra from that of isolated nitric acid in the gas phase^6,7,13,15–17,19^. Although structure distortion of HNO_3_ also occurs to some extent in the solution^14^, the quantum yield in aqueous nitrate photolysis is lower than 0.1 due to the recombination of the primarily-produced photo-fragments, e.g. NO_2_ and OH, before they leave the surrounding cage of HNO_3(s)_ - a so-called “cage” effect^20–23^. The quantum yield of HNO_3(s)_ photolysis was measured near unity in the laboratory^19,24^. The red-shift absorption spectra into the actinic region with increased photon density, absorption cross section enhancement and high quantum yield together lead to the enhanced photolysis rate constant of HNO_3(s)_.

Despite of the general information collected, detailed understanding on the kinetics and mechanisms of HNO_3(s)_ photolysis, e.g., how exactly the surface matrix affects HNO_3(s)_ photolysis, is still lacking. Organic compounds and HNO_3(s)_, are ubiquitous on the environmental surfaces. In this study, photolysis of HNO_3_ on Pyrex glass is investigated in a photochemical flow reactor over a wide range of HNO_3_ surface density (*D*_*HNO3*_), with or without model organic compounds in the surface matrix. The photolysis rate constant of HNO_3(s)_ are measured and described as a function of *D*_*HNO3*_ and the presence of the model organic compounds. The photolysis rate of HNO_3(s)_ is inhomogeneous as suggested by surface catalysis mechanism, and the influence of organic matters on the photolysis production of HONO and NO_2_ is discussed in the context of an integrated mechanism.

## Results

### Surface-catalyzed photolysis of HNO_3(s)_

Upon light exposure, production rates of HONO and NO_2_ (*P*_*HONO*_ and ${P}_{N{O}_{2}}$) increased immediately, followed by a rapid drop (non-exponential) in the initial few minutes and then a pseudo-exponential decay afterwards (Fig. S1). The signals nearly returned to the baseline level after the light was turned off. It was evidential that the immediate increase in *P*_*HONO*_ and ${P}_{N{O}_{2}}$ was in response to photolysis of HNO_3(s)_. The apparent or average photolysis rate constant of HNO_3(s)_ (*J*_*HNO3(s)*_) during the light exposure period is then determined by equation (Eqs 7–9) in the Method section and listed in Table 1. The rapid drop in the initial *P*_*HONO*_ and ${P}_{N{O}_{2}}$ suggests the inhomogeneity of photolysis reactivity of HNO_3(s)_, that is, some HNO_3(s)_ is more reactive than the rest^2,3^. It is proposed here that some HNO_3(s)_ molecules directly associated with surface reactive sites, thus acquire superior photochemical reactivity and tends to be photolyzed at much higher rates in the initial stage, relative to the rest, when exposed to the UV light.

**Table 1:** Summary of photolysis rate constant of HNO_3_ on Pyrex glass surface without and with model organic (~16 µmol m^−2^).

Surface Condition	*N*	Sub-monolayer HNO_3_: ~1.1 µmol m^−2^			Multilayer HNO_3_: ~25 µmol m^−2^		
		*j*_*HNO3*_−_*HONO*_ × 10^*−5*^ *s*^*−1*^	*j*_*HNO3*_−_*NO2*_ × 10^*−5*^ *s*^*−1*^	*J*_*HNO3*_ × 10^*−5*^ *s*^*−1*^	*j*_*HNO3*_−_*HONO*_ × 10^*−5*^ *s*^*−1*^	*j*_*HNO3*_−_*NO2*_ × 10^*−5*^*s*^*−1*^	*J*_*HNO3*_ × 10^*−5*^*s*^*−1*^
HNO_3_ only	5	1.6 ± 0.4	0.5 ± 0.2	2.1 ± 0.4	0.34 ± 0.03	0.17 ± 0.03	0.50 ± 0.01
Humic Acid*	2	3.4	0.8	4.3	0.4	0.1	0.5
Benzoic Acid	1	2.0	1.4	3.4	0.3	0.2	0.5
3-Hydroxybenzoic Acid	1	4.1	3.1	7.2	1.0	0.9	1.9
4-Hydroxybenzoic Acid	1	3.6	3.4	7.0	0.5	0.4	0.9
3-Hydroxybenzaldehyde	1	1.3	0.8	2.1	0.4	0.1	0.5
Salicylic Acid	2	19	9.7	28	2.5	0.9	3.4
Catechol	1	8.4	0.2	8.6	2.0	0.1	2.2
Resorcinol	1	5.8	0.3	6.2	0.7	0.1	0.8
Hydroquinone	1	25	1.9	27	4.3	0.7	5.0
Salicylic Acid + Hydroquinone	1	15	5.0	20	2.2	0.8	3.0
Citric Acid	2	9.1	2.4	11	1.6	0.2	1.8
Oxalic Acid	1	10	2.8	13	1.0	0.2	1.1
Succinic Acid	1	1.8	1.7	3.5	0.2	0.1	0.3
Ascorbic Acid	1	4.1	0.7	4.8	1.0	0.2	1.1
Glucose	1	2.4	1.9	4.3	0.3	0.1	0.4

^*^Humic Acid = 1.6 mg m^−2^.

The inhomogeneous photolysis reactivity of HNO_3(s)_ could be attributed to the surface catalysis effect on HNO_3(s)_ photolysis and be quantitatively described (Eqs 1–3)^3^. It is known that HNO_3_ distributes on the surface irregularly even under the “sub-monolayer” conditions^24^, with high affinity to the surface reactive sites^25^. The association of HNO_3(s)_ with surface reactive sites could lead to structure distortion of HNO_3(s)_ molecule, and thus change its absorption spectra and thus its photolysis reactivity^6,7,26^. In the proposed surface catalysis mechanism, the catalysis power depends on the nature of the surface reactive site and the type of the reaction, and it dissipates as more molecules are deposited on the upper layer and further away from the neighboring surface reactive site^27,28^. That is, for each infinitesimal amount of HNO_3(s)_, the corresponding photolysis rate constant (*j*) is an inverse function of surface coverage or surface density of HNO_3(s)_ (${D}_{HN{O}_{3}}$):

1 $${j}=\frac{a}{1\,+\,b\,{D}_{HN{O}_{3}}}+c$$

where *a, b* and *c* are the fitting constants. The constant *a* represents the maximum photolysis rate constant (s^−1^) for infinitesimally small amount of HNO_3(s)_ that is directly associated with the surface reactive sites. The constant *b* reflects the dissipation rate (m^2^ mol^−1^) of the catalysis power towards increased ${D}_{HN{O}_{3}}$, and *c* is the photolysis rate constant (s^−1^) of the upper-layer HNO_3(s)_ that is not closely associated with the surface reactive site. Then the total production of HONO and NO_2_ (*R*) from photolysis of all the HNO_3_ molecules on unit surface area is:

2 $$\begin{array}{rcl}R & = & {\int }_{0}^{{D}_{HN{O}_{3}}}jd({D}_{HN{O}_{3}})\\  & = & \frac{a}{b}\,\mathrm{ln}(1+b\,{D}_{HN{O}_{3}})+c\,{D}_{HN{O}_{3}}\end{array}$$

And the apparent or average photolysis rate constant of HNO_3(s)_ is the ratio of the production (*R*) to the amount of HNO_3(s)_ exposed to light on unit surface area (${D}_{HN{O}_{3}}$):

3 $$\begin{array}{rcl}{J}_{HNO3(s)} & = & \frac{R}{{D}_{HN{O}_{3}}}\\  & = & \frac{a}{b\,{D}_{HN{O}_{3}}}\,\mathrm{ln}(1+b\,{D}_{HN{O}_{3}})+c\end{array}$$

**Figure 1:**
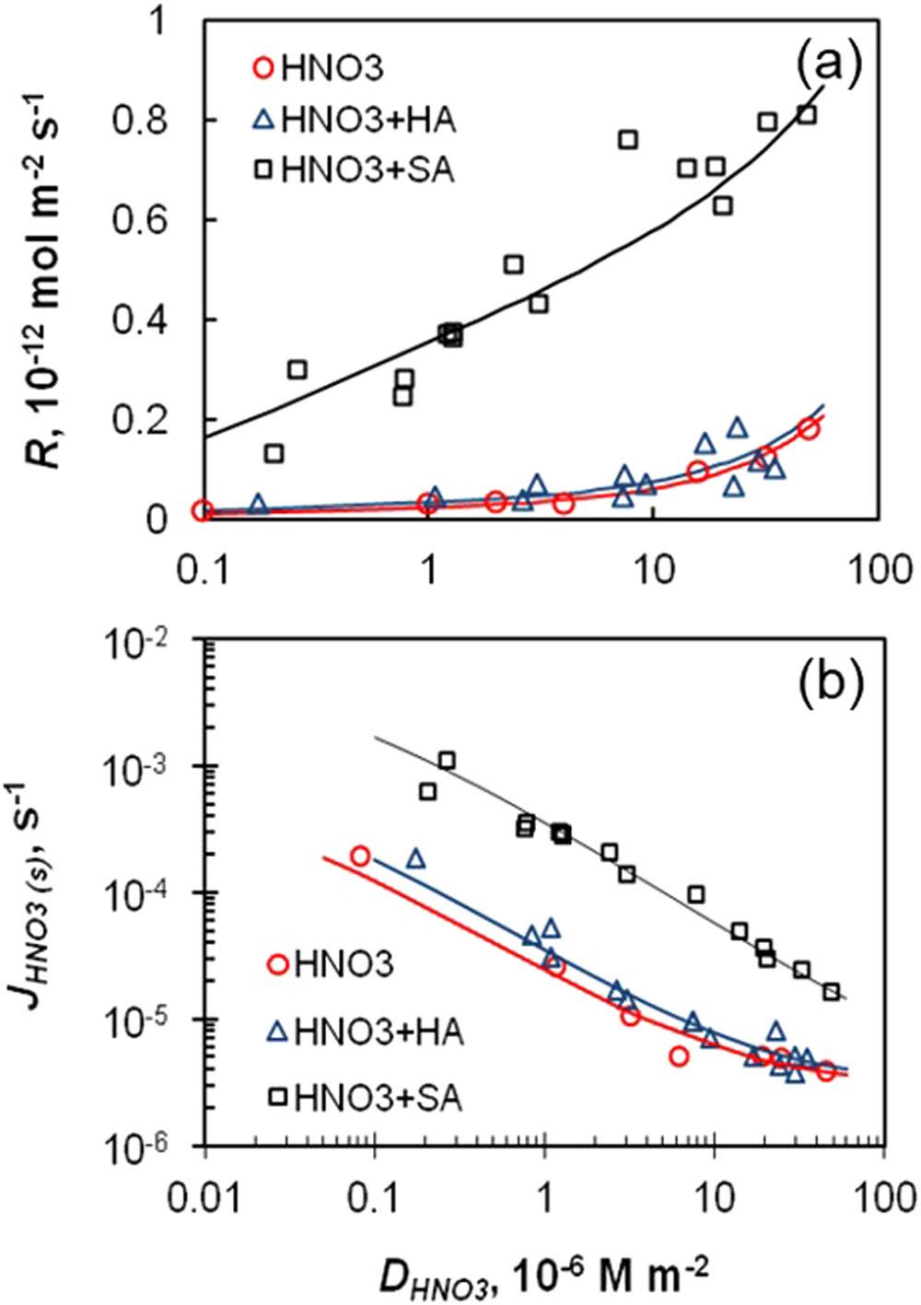
Relationships of total production rate of HONO and NO_2_ from HNO_3(s)_ photolysis (**a**) and the photolysis rate constant (**b**) with HNO_3_ surface density. The lines are the best fits to the data for equation (Eq. 2): the experiments of “HNO_3_” (, *a* = 6.5 × 10^−4^, *b* = 1.5 × 10^8^, c = 3.0 × 10^−6^, r^2^ = 0.96), “HNO_3_ + HA” (, *a* = 9.5 × 10^−4^, *b* = 1.5 × 10^8^, c = 3.0 × 10^−6^, r^2^ = 0.91), and “HNO_3_ + SA” (□, *a* = 5.0 × 10^−3^, *b* = 5.8 × 10^7^, c = 3.0 × 10^−6^, r^2^ = 0.93) and for equation (Eq. 8): the experiments of “HNO_3_ only” (, r^2^ = 0.95), “HNO_3_ + HA” (, r^2^ = 0.94), and “HNO_3_ + SA” (□, r^2^ = 0.98). The fitting constants *a*, *b*, and *c* are the same as in the two panels. HA and SA represent humic acid and salicylic acid, respectively.

The equation (Eq. 2) fits the experimental data well, with an r^2^ of 0.96 for “HNO_3_” experiments, an r^2^ of 0.91 for “HNO_3_ + HA” experiments, and an r^2^ of 0.94 for “HNO_3_ + SA” experiments (Fig. 1a). The equation (Eq. 3) again fits all the experimental data well with strong correlations (r^2^ values ≥ 0.94) (Fig. 1b). The higher *a* (5.0 × 10^−3^) and lower *b* (5.8 × 10^7^) values fitted for “HNO_3_ + SA” than those for “HNO_3_” suggest extra catalytic power of salicylic acid, in addition to the surface reactive site on Pyrex glass. This point is in good agreement with the substantial influence of salicylic acid on HNO_3(s)_ photolysis (see the next section). A single fitted *c* value of ~3 × 10^−6^ s^−1^ for all the three experiments represents a common photolysis rate constant of HNO_3(s)_ in the upper layer, where HNO_3_ complexed with water and itself, but is nearly unaffected by the catalysis power of surface reactive sites and the organic matters in the matrix^3^. A common *c* for all the experiments also seems reasonable, as all the experiments fitted in Fig. 1 were conducted under 50% RH, and HNO_3_-H_2_O complex in the upper layer would be the same.

In our previous paper on photolysis of ambient particulate nitrate and HNO_3_ deposited on natural and artificial surfaces, equation (Eq. 3) fits well those reported photolysis rate constants^2,3^. The good fitting of our data by Eq. 3 here and in our previous papers is quantitative evidence for the surface catalysis mechanism, and provides a robust parameterization method for the varied photolysis rate constant of HNO_3(s)_.

### Matrix effect of water and organic matters

**Figure 2:**
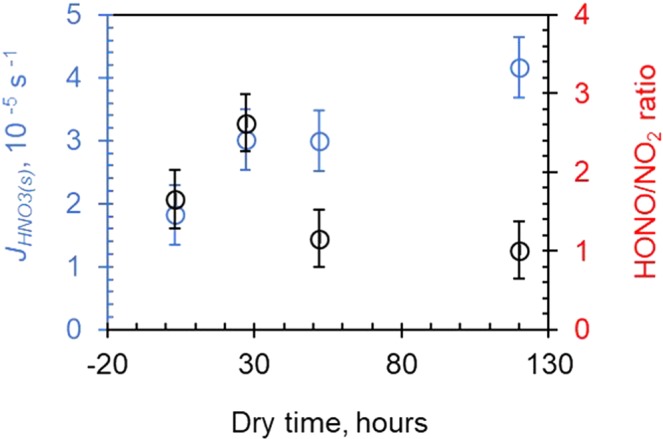
Plots of *J*_*HNO3(s)*_ and production ratio of HONO to NO_2_ as a function of dry time after coating the Pyrex glass surface with same amount of HNO_3_.

Water and organic matter are ubiquitous on the environmental surfaces and may have significant effects on both *J*_*HNO3(s)*_ and HONO/NO_2_ production ratio. As the surface -adsorbed water decreased slowly with the drying time of the sample, the *J*_*HNO3(s)*_ value was found to increase and the production ratio of HONO/NO_2_ to decrease (Fig. 2). This is consistent with a previous result, which shows higher *J*_*HNO3(s)*_ but lower HONO/NO_2_ production ratio at 0% RH than 50% RH^9^. Surface-adsorbed water could affect the photolysis reactivity of HNO_3(s)_ and HONO/NO_2_ production ratio via complex mechanisms^14,16,22,26^. At high RH, competitive adsorption of surface water with HNO_3(s)_ on surface reactive sites may reduce the apparent photolysis reactivity of HNO_3(s)_. Water cage potentially formed at high RH may further reduce the apparent photochemical reactivity by promoting the recombination of photo-fragments^20–23^, and favor the formation of HONO from reaction between primary product of NO_2_ and adjacent water as a H-donor. While at low RH, HNO_3_ and H_2_O cluster formed on the surface reactive site might enhance the photolysis reactivity of HNO_3(s)_^28^_._ The complex interaction of HNO_3(s)_ with co-absorbed H_2_O is consistent with varied absorption state and chemical environments of HNO_3(s)_, which determines the photolysis reactivity of HNO_3(s)_.

To investigate the matrix effect of organic matters on HNO_3(s)_ photolysis, 13 model organic compounds were chosen as proxies for the naturally occurring organics in the atmosphere (Table 1). Aromatic compounds were of special interest because they have absorption bands in the UVB (280–315 nm) actinic region as shown in the absorbance measurement of their water solution in Fig. S2 and may serve as photosensitizers. The isomers of hydroxybenzoic acids and benzenediols were examined and compared. Some non-aromatic organic acids and polyols were chosen to study matrix effects other than photosensitization because they generally do not absorb light in the UV actinic regions. Humic acid was selected due to its ubiquitous presence and its photosensitization effect in the photo-enhanced conversion of NO_2_ to HONO^29^. All these model organic compounds do not contain nitrogen and thus are not direct precursors to the target products of HONO and NO_2_.

Table 1 summarized the photolysis rate constant of HNO_3(s)_ at the presence of different model organic compounds. While multiple measurements were made for each model organic compound at different ${D}_{HN{O}_{3}}$, the table lists only the results at ${D}_{HN{O}_{3}}$ of ~1.1 × 10^−6^ mol m^−2^ and ~25 × 10^−6^ mol m^−2^ to represent the sub-monolayer and multilayer conditions, respectively. One or two of the model organic compounds was co-adsorbed with HNO_3(s)_ onto the Pyrex glass surface at a surface density of about 16 × 10^−6^ mol m^−2^ to form ~2 layers^3^, except for humic acid, which was coated at a surface density of 1.6 mg m^−2^.

In the absence of model organic compounds, a mean (±SD) *J*_*HNO3(s)*_ value of ~2.1 (±0.4) × 10^−5^ s^−1^ was measured for the sub-monolayer conditions, which was similar to a previous measurement value of 2.2 (±0.2) × 10^−5^ s^−1^ on an unpolished Pyrex glass surface at 50% RH^9^. Photolysis of HNO_3(s)_ is enhanced by the presence of all the model organic compounds relative to “HNO_3_” conditions; the magnitude of enhancement depends on both functional groups and substitution patterns of organic compounds.

**Figure 3:**
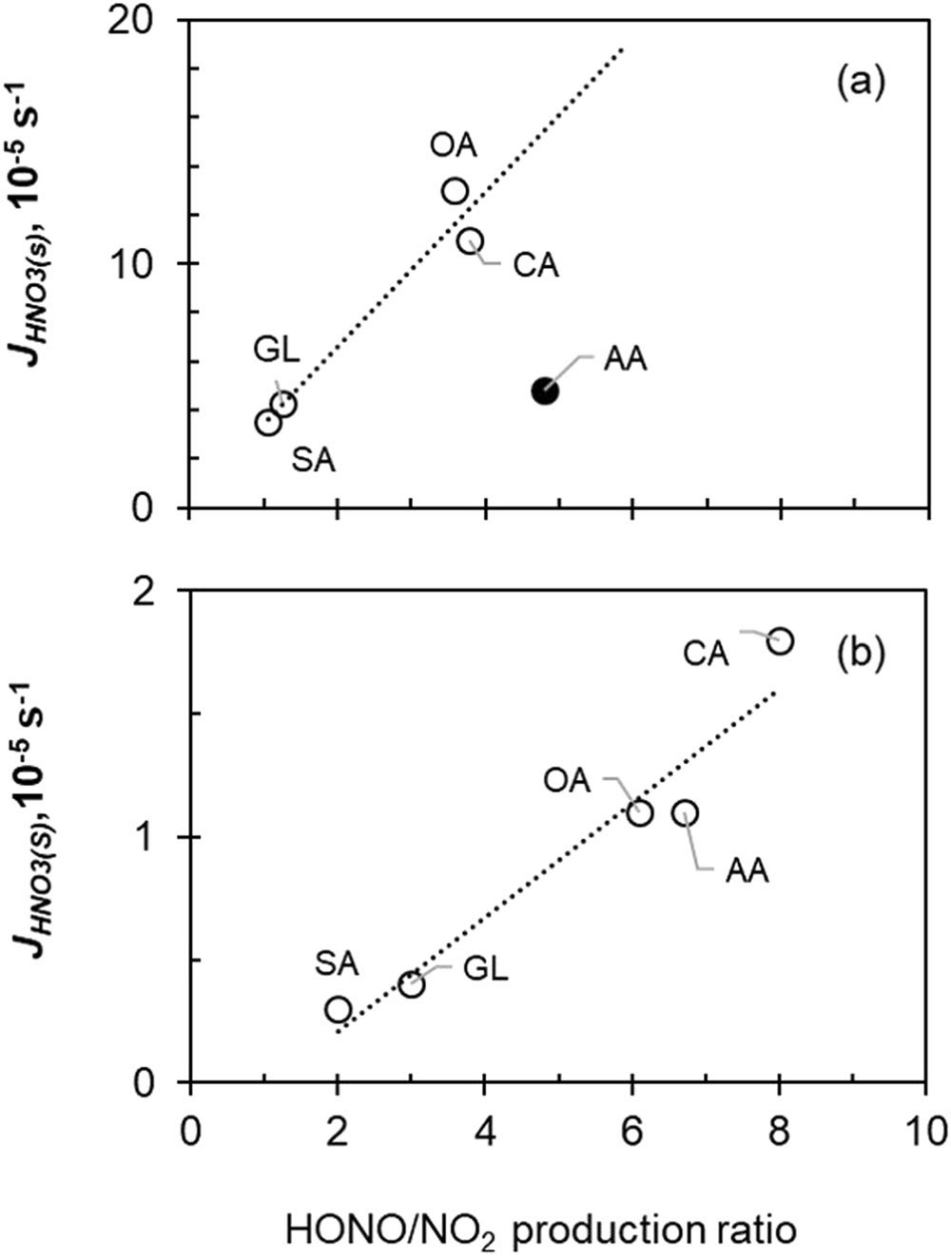
Correlation analysis between the *J*_*HNO3(s)*_ value and the production ratio of HONO/NO_2_ for non-aromatic compounds at a HNO_3_ surface density of ~1.1 × 10^−6^ mol m^−2^ (**a**) and 25 × 10^−6^ mol m^−2^ (**b**). OA, CA, GL, SA, and AA represent oxalic acid, citric acid, glucose, succinic acid and ascorbic acid, respectively. The solid circle represents an outlier datapoint of AA, which was not included in the fitting.

**Figure 4:**
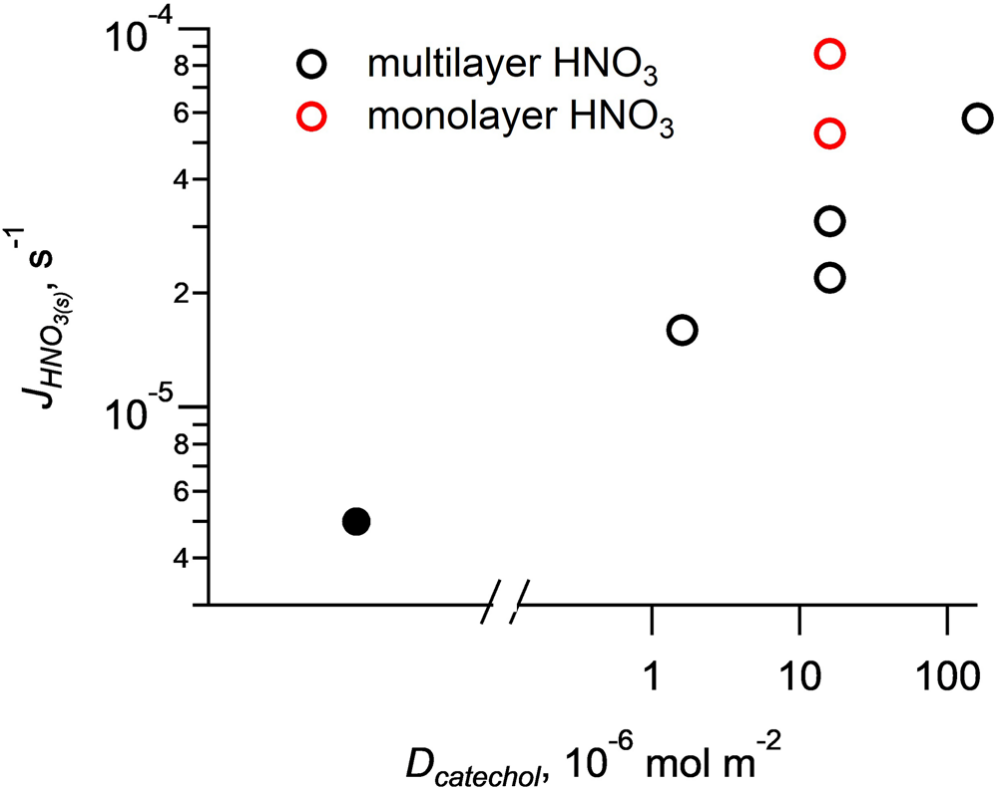
Log-log plot of the photolysis rate constant against catechol surface density. The photolysis rate constant increases with catechol surface density from data point for experiment without catechol (solid black cycle) to data points for increasing catechol surface density (black open cycle) Data points for ~ monolayer HNO_3_ (red open cycle) are above the data points for multilayer HNO_3_ (black open cycle) as catechol surface density is the same.

The presence of non-light-absorbing organic compounds significantly enhances the photolysis rate constant of HNO_3(s)_. Citric acid and oxalic acid enhance the ${J}_{HN{O}_{3}(s)}$ by a factor of 5–6, and succinic acid, ascorbic acid and glucose by a factor of ~2. The observed enhancement on HNO_3(s)_ photolysis by non-light-absorbing organic compounds is due to mechanisms other than photosensitization, such as direct participation in the reaction as H-donors^29,30^. All these model organic compounds, especially organic acids and polyols, are strong H-donors. They distribute with water molecules in the surrounding of HNO_3(s)_, weaken the water molecular “cage” and modify the chemical environment of HNO_3(s)_^19–22^. The reactions between these organic compounds and the initial photo-fragments of HNO_3(s)_ photolysis, i.e., OH and NO_2_, suppress the recombination of photo-fragments and thus enhance *J*_*HNO3(s)*_. The reactions between organic compounds and primary NO_2_ also produces secondary HONO and shifts the HONO/NO_2_ production ratio. ${J}_{HN{O}_{3}(s)}$ was then plotted against HONO/NO_2_ production ratio for both monolayer HNO_3_ and multiple-layer HNO_3_ experiments to test the hypothesis of H-donation reaction in HNO_3(s)_ photolysis (Fig. 3). Consistently strong correlation was found both in the monolayer HNO_3_ (Fig. 3a) and the multiple-layer HNO_3_ (Fig. 3b) experiments for organic acids and polyols, i.e., citric acid, oxalic acid, succinic acid, ascorbic acid and glucose (see Fig. S3 analysis to include more organic species). One exception was found in ascorbic acid in monolayer HNO_3_ experiment (Fig. 3a). Ascorbic acid is a strong reducing agent^31^; it may have reduced NO_2_ not just to HNO_2_ but further to NO, which was not detected by our NO_2_ measurement method, resulting in a lower apparent photolysis rate constant. To further test the H-donation hypothesis, different amount of catechol was co-absorbed with HNO_3_, and ${J}_{HN{O}_{3}(s)}$ was indeed found increased with catechol amount (Fig. 4). These two corroborative pieces of evidence in Figs 3 and 4 suggest the participation of H-donors in HNO_3(s)_ photolysis, affecting both *J*_*HNO3(s)*_ and the production ratio of HONO/NO_2_.

**Figure 5:**
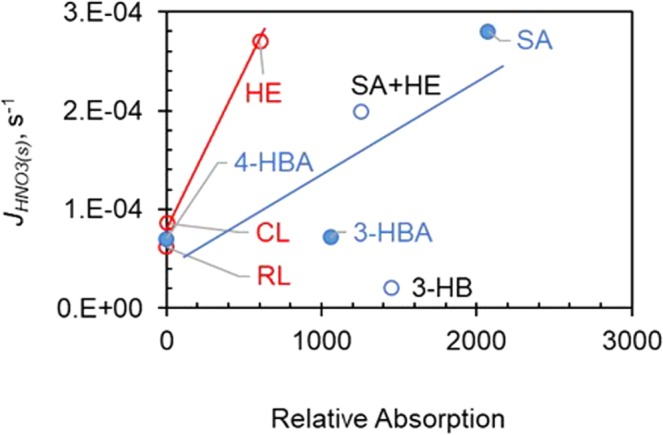
Correlation analysis between the enhanced *J*_*HNO3(s)*_ value and relative light absorption. The light with wavelengths below 300 nm was filtered by a Pyrex glass filter. HE, SA, 4-HBA, CL, RL, 3-HBA and 3-HB represent hydroquinone, salicylic acid, 4-hydroxybenzoic acid, catechol, resorcinol, 3-hydroxybenzoic acid and 3-hydrobenaldehyde, respectively.

The presence of light-absorbing organic compounds has an even stronger influence on the photolysis rate constant of HNO_3(s)_. Salicylic acid and hydroquinone exhibit highest enhancement effect on *J*_*HNO3(s)*_, by approximate one order of magnitude. Their isomers, i.e., 3-hydrobenzoic acid, 4-hydrobenzoic acid, catechol, and resorcinol only show modest enhancement effect, with enhancement factors of 3–4. Benzoic acid and humic acid also show small enhancement effect by a factor of ~2. To evaluate the photosensitization effect, the relative light absorption of organic compound solution (A) was calculated as the product of their absorption cross section from 290 nm to 360 nm and intensity spectra of the experimental light source at corresponding wavelength (Eq. 4).

4 $${\rm{A}}={\int }_{290\,nm}^{360\,nm}\,{\sigma }_{i}{\rho }_{i}d{\lambda }_{i}$$

where *σ*_*i*_ and *ρ*_*i*_ represent the absorption cross section of organic compounds and photon density of the light source as a function of wavelength. Within the same group of isomers (hydroxybenzoic acids or benzene-diols) photolysis rate is significantly enhanced by the light absorption (Fig. 5), suggesting the enhancement effect of photosensitization. However, no correlation between relative light absorption and the enhancement factor was found if all the tested organic compounds were considered (Fig. 5). Possible reason might lie in the fact that photosensitization reaction consists multiple primary steps, e.g., absorbing light, photo-electron generation, photo-electron transition and induction of final photolysis reaction. The yields of these steps are expected to vary with the structure of organic compounds and thus consistent relationship between light absorbance and photolysis rate would not be always expected. For example, despite of small difference in molecular structure of hydroxybenzoic acids and benzene-diols, they have demonstrated substantially different quantum yield as shown in the blue line and red line in Fig. 5. As such, the light absorption appears only a rather rough indicator of the photosensitization of organic compounds on HNO_3(s)_ photolysis (Fig. 5).

## Mechanisms

Photolysis of HNO_3_ on Pyrex glass surface is enhanced by 1–4 orders of magnitude compared to those in the aqueous solution and gas phases, depending on its surface density and the type of organic compounds coexisting in the surface (Table 1, Fig. 1b). The surface catalysis mechanism explains the observed enhancement on HNO_3(s)_ photolysis, and the derived Eq. 3 can fits the photolysis rate constants at different HNO_3_ surface density (Fig. 1), presenting Eq. 3 as a quite good parameterization method for HNO_3(s)_ photolysis.

In the catalysis mechanisms discussed above, HNO_3_ absorbs preferably on the surface reactive sites^25,26,32^. The association of HNO_3(s)_ with the surface reactive site distorts its molecular structure and results in enhanced absorption cross section and “red shift” of the absorption spectra into the actinic region^6,7,14,16,18,33^. Both changes in its absorption spectra contribute to the significant enhancement in the light absorption and the production of excited HNO_3(s)_, HNO_3_^*^_(s)_:

R1 $${{\rm{HNO}}}_{3({\rm{s}})}+h\nu \to {{\rm{HNO}}}_{3({\rm{s}})}^{\ast }$$

Organic compounds on the surface may associate with HNO_3(s)_, causing structure distortion and enhancement of the light absorption of HNO_3(s)_, analogous to the catalysis power of the surface reactive site, and could thus further increase the apparent yield of HNO_3_^*^_(s)_. This will result in a higher *a* value and a lower *b* value in the equations (Eq. 2-Eq. 3), for example, in “HNO_3_ + SA” experiments than in “HNO_3_” experiments (Fig. 1). In addition, organic chromophores are photosensitizers, which absorb light and transfer the energy to the adjacent HNO_3(s)_ and thus indirectly increase the apparent yield of HNO_3_^*^_(s)_.

The bond breakage of HNO_3_^*^_(s)_ produces OH_(s)_ and NO_2(s)_ as the dominant products (R2) and HONO as a minor product (R-2)^9,18,19^:

R2 $${{\rm{HNO}}}_{3({\rm{s}})}^{\ast }\leftrightarrow {{\rm{OH}}}_{({\rm{s}})}+{{\rm{NO}}}_{2({\rm{s}})}$$

R-2 $${{\rm{HNO}}}_{3({\rm{s}})}^{\ast }\to {{\rm{HONO}}}_{({\rm{s}})}+{\rm{O}}{({}^{3}{\rm{P}})}_{({\rm{s}})}$$

Certain fraction of these two photo-fragments, OH_(s)_ and NO_2(s)_, may recombine to form HNO_3_^*^_(s)_ (R-2)^20–23^, or react with organic and water molecules (R3–R5) to form new products including HONO.

R3 $${{\rm{OH}}}_{({\rm{s}})}+{{\rm{Org}}}_{({\rm{s}})}\to {\rm{Oxidized}} \mbox{-} {{\rm{Org}}}_{({\rm{s}})}+{{\rm{H}}}_{2}{{\rm{O}}}_{({\rm{s}})}$$

R4 $${{\rm{NO}}}_{2({\rm{s}})}+{{\rm{H}}}_{2}{{\rm{O}}}_{({\rm{s}})}\to {{\rm{OH}}}_{({\rm{s}})}+{{\rm{HONO}}}_{({\rm{s}})}$$

R5 $${{\rm{NO}}}_{2({\rm{s}})}+{{\rm{Org}}}_{({\rm{s}})}\to {\rm{Oxidized}} \mbox{-} {{\rm{Org}}}_{({\rm{s}})}+{{\rm{HONO}}}_{({\rm{s}})}$$

The water molecular cage effect could sufficiently decrease the quantum yield and thus photolysis rate constant^9^. The NO_2(s)_ produced from (R2) may possess excessive energy and may be more reactive than ground-state NO_2_ towards H donors (R4 and R5), leading to HONO production^31,34,35^. The H-donation reactions consume the photolysis fragments, prevent them from recombination (R-2), and thus enhance the quantum yield and *J*_*HNO3(s)*_^9,16,17^. Increase of *J*_*HNO3(s)*_ with HONO/NO_2_ production ratio (Figs 3 and S3) is consistent with the H-donation reaction mechanism (R4 and R5). The dependence of *J*_*HNO3(s)*_ on the abundance of a typical H donor, catechol, supports the argument that H donor directly participates in HNO_3(s)_ photolysis (R3–R5).

Finally, the produced NO_2_ from reaction (R2) and HONO from reactions (R-2, R4 and R5) release from surface to gas phase (R6 and R7):

R6 $${{\rm{HONO}}}_{({\rm{s}})}\to {{\rm{HONO}}}_{({\rm{g}})}$$

R7 $${{\rm{NO}}}_{2({\rm{s}})}\to {{\rm{NO}}}_{2({\rm{g}})}$$

Our measurement systems measured the released HONO and NO_2_ in the gas phase.

## Conclusion

The environmental surfaces, the deposited molecules of HNO_3_, organic matter and water form a complex system and play varied roles in HNO_3(s)_ photolysis, such as surface catalysis, photosensitization, and H-donation. An integrated photolysis mechanism is proposed here with detailed fundamental reactions and Eq. 3 to qualitatively and quantitatively describe the variation of *J*_*HNO3(s)*_, such as the dependence of *J*_*HNO3(s)*_ on HNO_3_ surface density, H donors (both water and organic compounds) and photosensitizer. To note, this mechanism investigation on the surface-catalyzed HNO_3_ photolysis in laboratory need to be supplemented with quantitative analysis on the photolysis rate constant and its dependence on ambient conditions, such as temperature. For that, further study should evaluate the kinetics and mechanisms of HNO_3_ photolysis in ambient conditions.

## Method

### Chemicals

All the chemicals were at least reagent grade or better, and were used without further purification, including: HNO_3_ (Ultrapure, J.T. Baker), Salicylic acid (ACS grade, Sigma-Aldrich), 3-hydrobenzoic acid (99%, Aldrich), 4-hydrobenzoic acid (ACS grade, Aldrich), catechol (≥99%, Sigma-Aldrich), resorcinol (ACS grade, Sigma-Aldrich), hydroquinone (≥99%, Sigma), citric acid (ACS grade, Sigma-Aldrich), oxalic acid (≥99%, Sigma-Aldrich), succinic acid (≥99%, Sigma-Aldrich), ascorbic acid (reagent grade, Sigma-Aldrich), Benzoic acid (99%, Aldrich), glucose (≥99.5%, Sigma), humic acid sodium salt (Technical grade, Aldrich), NaNO_2_ (99.7%, J.T. Baker), NaNO_3_ (99.9%, J.T. Baker), sulfanilamide (SA) (≥99%, Aldrich), N-(1-Naphthyl) ethylene-diamine (NED) (ACS grade, Aldrich), NaOH (99.99%, Aldrich), NH_4_Cl (99.99%, Aldrich), HCl (Aldrich), sodium benzoate (99%, Aldrich), and sodium salicylate (≥99%, Sigma-Aldrich). Water was purified with a Barnstead Nanopure Diamond system or Millipore Milli-Q water system, with resistivity ≥18.2 MΩ.

### Experimental Setup

The light exposure experiment was conducted in a cylindrical Teflon flow reactor with a quartz window on the top. The diameter is 10 cm and depth is 2.5 cm, with a cell volume of ~200 ml. A sandblasted borosilicate glass (Pyrex) surface (~9 cm diameter) was used as the “substrate” for coating HNO_3_ and organic compounds, and was placed in the flow reactor for light exposure. Ultra-high-purity nitrogen (Airgas, UHP200) was used as the carrier gas. The carrier gas flowed through a thermostatic water bubbler at 9.2 ± 0.1 °C at a flow rate of 450 ml min^−1^ and then went through a long thermal-equilibration coil at the room temperature of 21 ± 1 °C. The RH of the carrier gas entering the flow reactor was then at 50 (±2) %. The gaseous products were sampled by two coil samplers connected in series at a gas flow rate of 400 mL min^−1^. Purified water was used to scrub HONO in the first 10-turn coil sampler at 100% efficiency. A reagent solution containing 60 mM sulfanilamide (SA) and 0.8 mM N-(1-Naphthyl) ethylene-diamine (NED) in 2.5 M acetic acid was used to scrub NO_2_ in the second 40-turn coil sampler at 60% efficiency. The remaining outflow of ~50 ml min^−1^ from the reactor was vented through a 10-cm 1/16” ID Teflon tubing to maintain slightly positive pressure in the reactor. All tubing used in the cabinet was wrapped by aluminum foil to shield from the UV light. The collected HONO and NO_2_ were derivatized by SA and NED and analyzed as azo dye by two long-path absorption photometric (LPAP) systems^36^. Each LPAP system consists of a miniature fiber optic spectrometer (USB2000, Ocean Optics), a 1-m liquid-waveguide capillary flow cell (LWCC-3100, WPI) and a tungsten light source (FO-6000, WPI). The detection limits for HONO and NO_2_ are 6 pptv and 15 pptv, respectively.

A 450-watt medium pressure mercury arc lamp (ACE Glass, model 7825) was placed 20 cm above the reactor as the light source. The light was filtered by a Pyrex sleeve to remove low wavelength UV light (<290 nm) and by a quartz well filled with circulated water to remove heat-generating infrared light. Temperature in the photochemical reactor increased slightly during light exposure, by 1–2 °C. Effective UV intensity was monitored using a nitrate actinometer^34^.

### Surface preparation

The Pyrex glass surface was first cleaned thoroughly with Micro-90 cleaning solution (Cole-Parmer), and then repeatedly rinsed by ethanol and DI water. The cleaned Pyrex glass surface was dried in a vacuum desiccator before applying surface coating.

Thirteen model organic compounds with and without chromophores are chosen as proxies for the naturally occurring organic matters in the atmospheric environment. HNO_3_ with or without model organic compounds were coated onto Pyrex glass surface by applying 0.1 ml coating solution of known concentrations, and spreading the solution out uniformly with a hydrophobic Teflon blade. Organic solutions were made from their corresponding sodium salts for better water solubility and were acidified to pH ~4 with 1 M H_2_SO_4_ before being mixed with HNO_3_ solution. HNO_3_ mainly partitions in the NO_3_^−^ form at pH value of ~4. The coated surface with or without model organic compounds was allowed to dry overnight in the vacuum desiccators before use in the light exposure experiments.

To quantify the amount of HNO_3_ exposed to light, the coated HNO_3_ was carefully rinsed off from the Pyrex glass surface with 10 ml 1% NH_4_Cl buffer solution (pH = 8.5). The wash solution was analyzed immediately in a LPAP system with a copperized Cd-column to convert nitrate into nitrite^37^. HNO_3_ surface density was then calculated from the determined HNO_3_ amount and the geometric area of the Pyrex glass surface (62 cm^2^). A range of HNO_3_ surface density of (0.1–80) × 10^−6^ mol m^−2^ was used to simulate sub-monolayer and multilayer conditions^38^. To account for the light absorption of organic compounds, absorbance spectra in the wavelength of 200–600 nm of the coating solution of HNO3 and organic compounds with a pH value around 4 were scanned using a UV-visible spectrometer (JENWAY, model 6405) with 1-cm path length (Fig. S2).

### Background level and corrections

Several corrections are made when calculating HONO and NO_2_ production rates and photolysis rate constants. Background signals from dark experiment were used as experiment baseline and were subtracted from the light exposure signals. Blank signals contributed from photolysis of deposited HNO_3_ on reactor and window surface were corrected by subtracting one half of the blank signals from the light exposure signals, since the bottom of the flow reactor was covered by the Pyrex glass surface in light exposure experiment. Photolytic losses of HONO and NO_2_, the products from HNO_3(s)_ photolysis, in the flow reactor during light exposure experiment were also considered. With a residence time of about 30 seconds in the flow reactor, ~5% HONO loss was calculated, and about 25% NO_2_ loss was observed when a gaseous NO_2_ standard (Matheson Tri-Gas Inc., CP) was introduced into the flow reactor.

The production rate (nmol s^−1^) of HONO, *P*_*HONO*_, and production rate of NO_2_, ${P}_{N{O}_{2}}$, were calculated by:

5 $${P}_{HONO}=\frac{{C}_{si}^{HONO}-{C}_{ri}^{HONO}/2}{60\times 1000}\times {F}_{l}^{HONO}\times \frac{450}{400}\times \frac{1}{0.95}\,$$

6 $${P}_{N{O}_{2}}=\,\frac{{C}_{si}^{NO2}-{C}_{ri}^{NO2}/2}{60\times 1000}\,\times {F}_{l}^{NO2}\times \frac{450}{400}\,\times \frac{1}{0.6\times 0.75}\,$$

where *C*_*si*_ and *C*_*ri*_ are the concentrations (nM) of HONO or NO_2_ measured in the scrubbing solutions during the light exposure and the blank control experiments, respectively; F_l_ is the scrubbing solution flow rates at 0.24 ml min^−1^ and 0.4 ml min^−1^ for HONO and NO_2_, respectively; the ratio of $\frac{450}{400}$ is the correction for minor overflow loss from the reactor; the coefficient of 0.6 is the collection efficiency in the NO_2_ channel; the coefficients of 0.95 and 0.75 are the corrections for photolysis losses of HONO and NO_2_, respectively.

The photolysis rate constants (s^−1^) of HNO_3_ leading to productions of HONO (${j}_{HN{O}_{3}\to HONO}$) and NO_2_ (${j}_{HN{O}_{3}\to N{O}_{2}}$), and the overall photolysis rate constant of HNO_3_ (${J}_{HN{O}_{3}(s)}$) on the surface were determined by equations (Eqs 6–8):

7 $${j}_{{{\rm{HNO}}}_{3}\to {\rm{HONO}}}\,=\frac{{{\rm{P}}}_{{\rm{HONO}}}\,}{{{\rm{N}}}_{{{\rm{HNO}}}_{3}}}\times \frac{3.0\times {10}^{-7}}{{J}_{nitrate}}$$

8 $${j}_{{{\rm{HNO}}}_{3}\to {{\rm{NO}}}_{2}}=\frac{{{\rm{P}}}_{{{\rm{NO}}}_{2}}}{{{\rm{N}}}_{{{\rm{HNO}}}_{3}}}\times \frac{3.0\times {10}^{-7}}{{J}_{nitrate}}$$

9 $${J}_{HN{O}_{3}(s)}={j}_{HN{O}_{3}\to HONO}+{j}_{HN{O}_{3}\to N{O}_{2}}$$

where ${{\rm{N}}}_{{{\rm{HNO}}}_{3}}$ is the amount of HNO_3_ exposed to light; ${J}_{nitrate}$ is the photolysis rate constant of nitrate in the actinometer solution exposed to the light source^34^. The calculated photolysis rate constants from Equations (Eqs 3–8) have been normalized to tropical noontime conditions on the ground (Solar elevation angle ϴ = 0°) where photolysis rate constant is ~3 × 10^−7^ s^−1^ for aqueous nitrate and ~7 × 10^−7^ s^−1^ for gaseous HNO_3_^34^. It should be pointed out that the calculated photolysis rate constant ${J}_{HN{O}_{3}(s)}$ in equation (Eq. 8) is based on the productions of HONO and NO_2_, the two dominant products. It may underestimate the true photolysis rate constant of HNO_3_ on the surface if other products are also produced and not corrected for. Production of NO from HONO and NO_2_ photolysis has been corrected in equations (Eq. 4) and (Eq. 8). The reproducibility of results from repeated experiments was better than 30%. The overall uncertainty of the measurement was determined to be within 50%, considering the uncertainty contributed by measurements of HONO, NO_2_, HNO_3(s)_ amount, gas phase flow rate, liquid phase flow rate and effective light intensity.

## Supplementary information

SI
