# Supplementary material for: Matrix effect on surface-catalyzed photolysis of nitric acid
Source: Sci Rep. 2019 Mar 13;9:4351. doi: 10.1038/s41598-018-37973-x (PMC6416357; doi:10.1038/s41598-018-37973-x)
Supplement: Supplementary file 1 — SI [file 41598_2018_37973_MOESM1_ESM.pdf]

Matrix effect on surface-catalyzed photolysis of nitric acid

Chunxiang Ye[1,2*], Ning Zhang[2], Honglian Gao[2], and Xianliang Zhou[2,3*]

[1]State Key Joint Laboratory of Environmental Simulation and Pollution Control, College of Environmental Sciences and Engineering, and Center for Environment and Health, Peking University, Beijing 100871, China

[2]Wadsworth Center, New York State Department of Health, Albany, NY 12201

[3] Department of Environmental Health Sciences, State University of New York, Albany, NY 12201

[*] Correspondence to: C. Ye (c.ye@pku.edu.cn) and X. Zhou (xianliang.zhou@health.ny.gov)

Supplementary information

As all the organic compounds was examined for this correlation, they were located in three groups (Figure S3). Moderate correlation was also found for four aromatic compounds, i.e., 4-hydroxybenozic acid, benzoic acid, catechol and resorcinol. Careful review of the absorption spectra of these four aromatic compounds reveals that they only weakly absorb light in 300 -360 nm region. In contrast, the rest of the aromatic compounds, i.e., salicylic acid, 3-hydroxybenzoic acid, hydroquinone, show considerable light absorption in 300-360 nm region, and no consistent correlation was found for them. It is proposed here that co-existing photosensitization effect with the H-donating reaction explain the moderate or even non-consistent correlation for the latter two group of organic compounds.

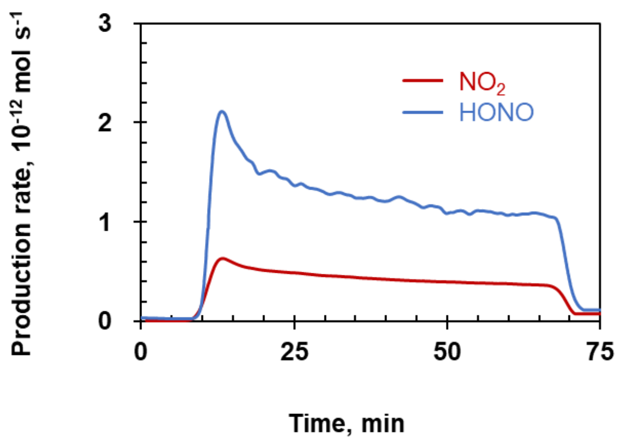

Figure S1 Time series of production rates of HONO and NO_2 from photolysis of 20 × 10^{-6} mol m^{-2} HNO_3 with 16 × 10^{-6} mol m^{-2} salicylic acid on Pyrex glass surface.

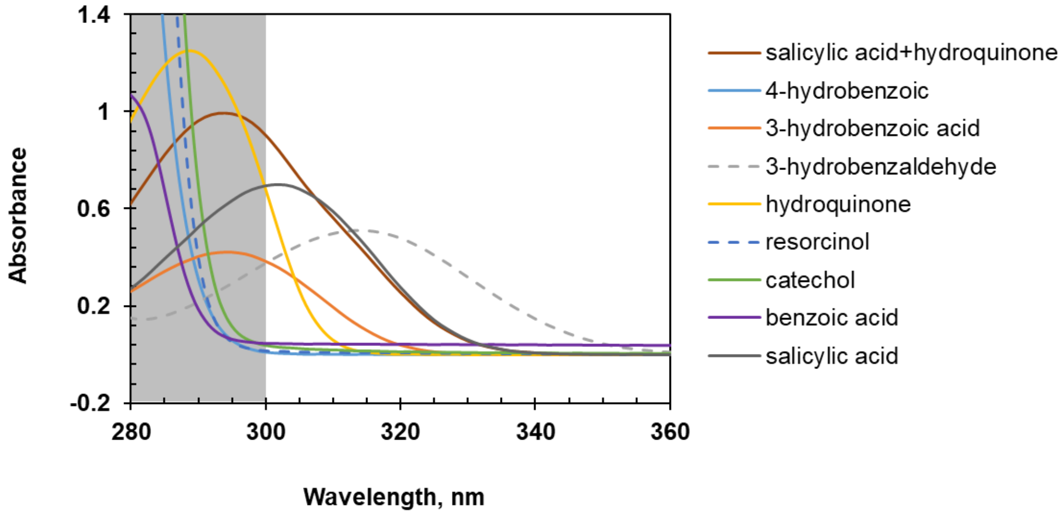

Figure S2 Absorbance of the coating solution of HNO_3 and different organic compounds with aqueous pH value around 4. The shade area indicates the light removed from the light source by a Pyrex glass filter with a cut-off wavelength of ~ 300 nm.

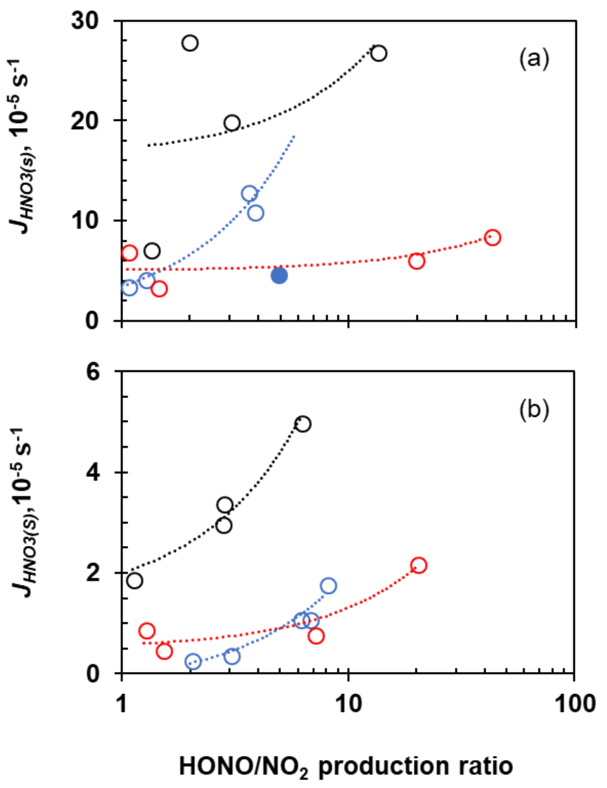

Figure S3 Correlation analysis between the enhanced $J_{HNO3(s)}$ value and the production ratio of HONO/NO_2 for 13 organic compounds at a HNO_3 surface density of ~1.1×10^{-6} mol m^{-2} (a) and 25×10^{-6} mol m^{-2} (b). All organic compounds fall into three group： non-aromatic compounds (blue cycle), non-chromophore aromatic compounds (red cycle) and chromophores (black cycle). The solid blue circle represents an outlier datapoint of AA, which is not included in the fitting. The x-axis was in log-scale for clarity.
